# A meta-analysis of chemokines in vitiligo: Recruiting immune cells towards melanocytes

**DOI:** 10.3389/fimmu.2023.1112811

**Published:** 2023-02-24

**Authors:** Reinhart Speeckaert, Arno Belpaire, Marijn M. Speeckaert, Nanja van Geel

**Affiliations:** ^1^ Department of Dermatology, Ghent University Hospital, Gent, Belgium; ^2^ Department of Nephrology, Ghent University Hospital, Gent, Belgium

**Keywords:** vitiligo, chemokine, disease activity, biomarker, CXCL

## Abstract

Chemokine research offers insightful information on the pathogenesis of cutaneous immune disorders, such as vitiligo. Compared to cytokines, the higher detectable levels of chemokines display promising potential as future disease biomarkers. Nonetheless, some published study results are contradictory, which can be attributed to patient characteristics and methodological differences. In this study, a meta-analysis was performed to compare chemokine expression in blood and skin samples from vitiligo patients versus healthy controls. Furthermore, the relationship between chemokine expression and disease activity was evaluated. Chemokine levels were investigated in 15 articles in the circulation and in 9 articles in vitiligo skin. Overall, some clear trends were observed. CXCR3 signaling by CXCL10 and CXCL9 has been confirmed by several reports, although CXCL10 showed more robust findings in blood samples. In this meta-analysis, CCL5, CXCL8, CXCL12, and CXCL16 levels were also significantly elevated. This indicates a complex immune pathway activation in vitiligo that overall supports a Th1-dominant response. Chemokines linked to the Th2 and Th17 pathways were less prevalent. Despite these findings, study protocols that examine a broader range of chemokines are encouraged, because current research is mostly focused on a small number of chemokines that were differentially expressed in previous studies.

## Introduction

Chemokines are signaling proteins regulating the recruitment of immune cells to the skin. Currently, around 50 chemokines and 18 receptors have been identified, often with overlapping functions leading to redundancy (1). Interestingly, different chemokines are involved in unfolding specific innate and targeted immune responses encountered in inflammatory skin disorders. Chemokines show great potential as disease biomarkers and provide important data to unravel the pathogenesis of complex immune responses. Chemokine levels are generally higher than cytokines allowing for more robust and reproducible results. Their short half-life ensures that changes can be detected rapidly. However, this trait may also introduce bias due to temporary infectious diseases (e.g., viral infections) which may cloud the chronic level of inflammation in patients with autoimmune disorders (2, 3).

In recent years, a lot of research has been carried out on chemokines in vitiligo allowing for rapid progress in this field. Unraveling the signaling pathways facilitates the development of targeted therapies (2). In this study, we performed a meta-analysis of the published data on chemokines in the circulation and the skin of vitiligo patients.

## Methods

A systematic search was done in PubMed and Embase to detect all articles investigating chemokines in vitiligo and comparing the values of vitiligo patients with healthy controls and/or active versus stable vitiligo patients. All articles from inception to October 2022 were eligible. The search was done between October 13^th^, 2022, and October 20^th^, 2022. The search strategy included the keywords ‘chemokine’ AND ‘vitiligo’ in all fields. The Prisma Flow Diagram can be found in the **Supplementary Material** (**Supplementary Figure 2**). Only human studies investigating chemokine levels in the blood and/or skin of vitiligo patients were included. Articles investigating animal models and abstracts were excluded. The aim was to investigate the difference in circulating and (peri-)lesional chemokine levels between vitiligo patients and healthy controls as well as between active and stable vitiligo patients. Results comparing chemokine levels before and after treatment were excluded because they may not be representative of the comparison of active versus stable disease. Meta-analysis was done in the case at least 2 articles performed an analysis of chemokines at the protein level using the same methodology. The meta-analysis was done with Review Manager 5.4.1 (The Cochrane Collaboration, 2020) using an inverse variant random effects model with the standardized mean difference as an effect measure. The standardized mean difference instead of the mean difference was used given the overall high variety in baseline chemokine concentrations in healthy controls pointing to an important difference in the results depending on the used lab kit and technology for measuring chemokine concentrations. When the difference between healthy controls and stable patients was less than 10%, the mean difference was used because this indicates that the methodology in these studies is likely comparable. The mean chemokine concentrations, standard deviation, and number of patients were extracted from each publication. When only the sample size, median, range, and/or interquartile range were provided, the mean was calculated based on the formula of Luo et al., 2018 and the standard deviation was based on Wan et al., 2014 (4, 5). For studies only displaying the results in graphs, data were extracted with GIMP 2.10.30 (GNU image manipulation program) using the method described by Van der Mierden et al., 2020 (6).

## Results

21 articles were identified that compared the chemokine levels in vitiligo patients versus controls and/or between active and stable vitiligo patients (**Supplementary Figure 1**). Most results on chemokines in the circulation were found for CXCL10 (n=15), followed by CXCL9 (n=11), CCL5 (n=5), CXCL12 (n=4), CXCL8 (n=3), CXCL11 (n=4), CCL2 (n=2), CCL1 (n=1), CXCL1 (n=1), CCL3 (n=1), CCL4 (n=1), CXCL13 (n=1), CXCL16 (n=2), CCL17 (n=1), CCL20 (n=2), CCL22 (n=1), CCL27 (n=1), and CCL28 (n=1). Chemokine levels in the skin were measured in 10 studies for CXCL10 (n=9), CXCL9 (n=7), CXCL11 (n=4), CCL5 (n=2), CCL20 (n=2), CXCL8 (n=1), CCL17 (n=1), CCL22 (n=1).

## Chemokines attracting CD8+ cytotoxic T lymphocytes

### CXCR3 binding ligands: CXCL9, CXCL10 and CXCL11

Multiple chemokines can bind to the CXCR3 receptor leading to apparent functional redundancy (7). Three isoforms of CXCR3 exist: CXCR3-A, CXCR3-B and CXCR3-alt. CXCL9 and CXCL10 bind to CXCR3-A and CXCL9, CXCL10, CXCL11, CXCL4 bind to CXCR3-B (8). It should be noted that chemokine ligands binding to the same receptor can still exert a specific effect. due to differences in geographical expression leading to a tissue-specific role, and differences in timing of expression. The non-redundant roles of CXCL9, CXCL10 and CXCL11 have been confirmed by different reports (9, 10). CXCR3A is the predominant form produced by lymphocytes although CXCR3B expression has also been found in CXCL10 interferon-activated Th1 lymphocytes, CD8 T cells, NK cells, NKT cells, dendritic cells, and some endothelial cells (7). Stimulation of melanocytes by IFN-γ leads to the production of CXCL9, CXCL10, and to a lesser amount of CXCL11 and CXCL4 (11). Melanocytes express CXCR3B, although the exact levels of CXCR3-A and CXCR3-alt expression have not been determined. CXCR3 expression is elevated in vitiligo melanocytes compared to healthy pigment cells. Keratinocytes express also CXCR3, although this appears less prominent (11).

### CXCL9

**Figure 1:**
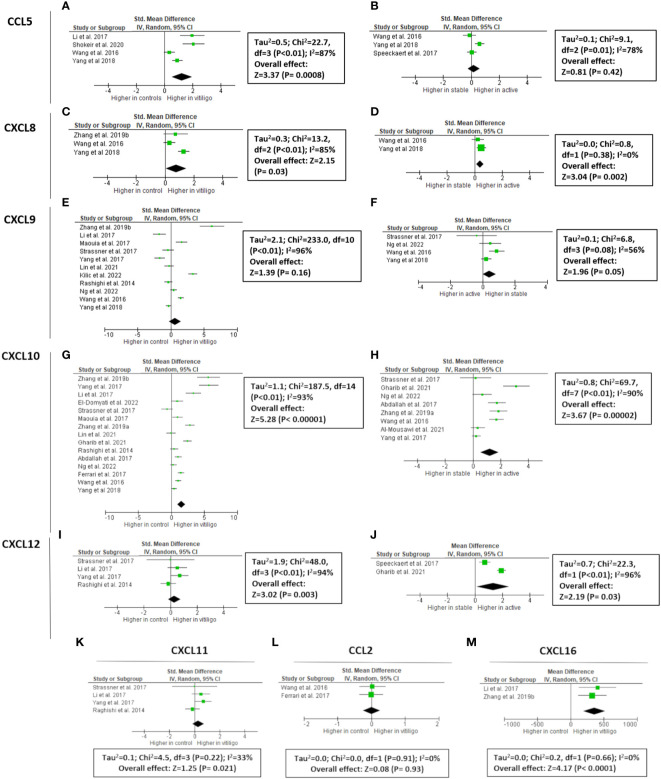
Meta-analysis of circulating chemokines of vitiligo patients versus controls or active versus stable patients for CCL5 **(A**, **B**, respectively), CXCL8 (**C**, **D**, respectively), CXCL9 (**E**, **F**, respectively), CXCL10 (**G**, **H**, respectively), CXCL11 **(K)**, CXCL12 (**I**, **J**, respectively), CCL2 **(L)**, and CXCL16 **(M)**. CCL: chemokine (C-C motif) ligand; CXCL: chemokine (C-X-C motif) ligand.

CXCL9 expression is upregulated by IFN-γ and in contrast to CXCL10 and CXCL11 not by IFN-α/β (12). CXCL9 has been extensively studied in vitiligo (497 vitiligo patients vs 257 healthy controls), although the data have been variable (**Figure 1**). 4/11 studies mentioned increased CXCL9 concentration in the circulation of vitiligo patients compared to healthy controls (13–23). Meta-analysis showed an overall non-significant result (P = 0.16). Only 1/5 reports found elevated circulating CXCL9 levels in active compared to stable vitiligo patients, although the final results of studies investigating CXCL9 in blister fluid were borderline significant (P = 0.05). CXCL9 is overexpressed in vitiligo skin compared to healthy skin (5 studies: 2 blister fluid, 2 mRNA of the skin, 1 immunohistochemistry) (17–19, 23, 24). A dramatic increase in CXCL9 concentrations in the actively progressing lesions compared to stable vitiligo skin became apparent from 2 studies (5.3-12.3 fold increase; 19 active vs 36 stable; concentrations in blister fluid) (16, 18).

### CXCL10

Vitiligo melanocytes have a higher constitutive CXCL10 production compared to healthy controls (11). A total of 709 patients and 362 controls were investigated in 15 studies. 12/15 studies (80%) reported significantly higher CXCL10 levels in vitiligo patients compared to controls, resulting in an overall highly significant CXCL10 concentration in the blood of vitiligo patients (P < 0.00001) (13–20, 22, 23, 25–28). The difference between patients and controls varied in the different studies with one study reporting a 7.8-fold increase (15). The largest study by Yang et al., 2018 (160 vitiligo patients versus 40 controls) found only a 1.3-fold increase (13). The baseline values of controls and vitiligo patients were very different depending on the publication. The mean values of CXCL10 ranged from 21.5 pg/ml to 1619 pg/ml in healthy controls and 40.09 pg/ml to 916.9 pg/ml in vitiligo patients. Although the higher expression of CXCL10 was confirmed by 12/15 studies, it is impossible to reliably estimate the magnitude of the difference due to the marked variety between study results. 8 studies compared active versus stable vitiligo patients, with 5 pointing to significantly increased serum CXCL10 concentrations in case of disease activity. This resulted in a P value = 0.0004 by meta-analysis. 6 studies analyzed CXCL10 expression in the skin with a comparison between vitiligo patients and healthy controls: 3 analyzing CXCL10 concentrations in blister fluid, 2 mRNA of the skin and 1 evaluating skin biopsies by immunohistochemistry (**Supplementary Figure 2**) (13, 17–19, 26). All findings resulted in a higher CXCL10 expression in vitiligo compared to healthy skin. 3 studies analyzed the chemokine concentration in blister fluid of active versus stable vitiligo skin, also pointing to dramatically increased values in progressive vitiligo lesions (3.04-11.7 fold increase) (13, 16, 18).

### CXCL11

Compared to CXCL9 and CXCL10, the expression of CXCL11 seems less pronounced in skin diseases. Some epidermal expression has been documented in CDLE and dermal expression in Jessner’s lymphocytic infiltration (29). CXCL11 is induced by IFN-γ, IFN-β, and to a lesser extent by IFN-α. In contrast to CXCL10, CXCL11 induces the polarization of IL10-secreting T-regulatory type 1 cells, restraining autoimmunity (1). CXCL11 has the highest affinity for CXCR3 compared to CXCL10 and CXCL9 (12). In vitiligo, CXCL11 has little data including 4 studies with in total of 62 vitiligo patients and 67 controls (17, 18, 20, 23). The overall meta-analyses detected no significant increase in vitiligo patients (P = 0.21). Two studies found no clearly increased mRNA CXCL11 levels in vitiligo skin, while one study did (17, 18, 23).

### CXCL16/CXCR6 receptor

Circulating CXCL16 levels were elevated in vitiligo in 2 studies (40 vitiligo patients and 24 healthy controls) (15, 20). Oxidative stress (H_2_O_2_) is correlated with CXCL16 mRNA levels in the skin of progressive vitiligo patients and serum CXCL16 decreases following successful therapy (15, 20). CXCL16 is produced by keratinocytes by ROS *via* an unfolded protein response. CXCL16-CXCR6 signaling regulates the recruitment of CD8+ T cells to vitiligo skin (20).

## Chemokines recruiting both innate and adaptive immune cells

### CXCL12: A CXCR4 binding chemokine

CXCL12 binds to two receptors, namely CXCR4 and ACKR3. CXCL12 is also known by its former name stromal cell-derived factor-1α (SDF-1α). It plays a homeostatic role in many tissues but has also a proinflammatory role and assists in T lymphocyte homing. Additionally, the recruitment of neutrophils, immature DCs, macrophages, innate lymphoid cells, and NK cells is promoted by CXCL12 (30). CXLCL12 can induce the expression of iNOS in CD8 T lymphocytes (31). iNOS is increased in vitiligo lesions and has been linked to melanocyte destruction (32). All 4 studies (177 vitiligo patients versus 82 controls) on circulating CXCL12 in vitiligo reported increased values in vitiligo patients compared to controls, resulting in a highly significant P-value of the meta-analysis (P = 0.003) (15, 19, 25, 33). Compared to other chemokines, the reported concentrations were relatively similar between the different reports. CXCL12 concentrations are also 1.2-1.7 higher in active vitiligo (2 studies; 45 active vs 65 stable) (25, 33).

Elevated CXCL12 expression has been demonstrated in many inflammatory and autoimmune skin diseases including psoriasis, rheumatoid arthritis, systemic lupus erythematosus, and inflammatory bowel disease. Furthermore, CXCL12 antagonists can delay disease onset and/or disease progression in inflammatory disorders (34). Melanocytes carry CCR4 and produce CXCL12 upon stimulation with LPS (35). Vitiligo melanocytes exhibited a 55.3 times upregulation based on RNA analysis compared to normal melanocytes (36). CXCL12 is one of the top secretary proteins upregulated in vitiligo (37). Additionally, CXCL12 regulates the migration and differentiation of melanocyte precursors and may play a role in the repigmentation of vitiligo lesions. On the other hand, some authors have hypothesized that CXCL12 may be responsible for hair graying or poliosis by the migration of melanocyte precursors out of the immune protective environment of the hair bulb (38, 39).

### CCL5/RANTES: CCR5 signaling

**Figure 2:**
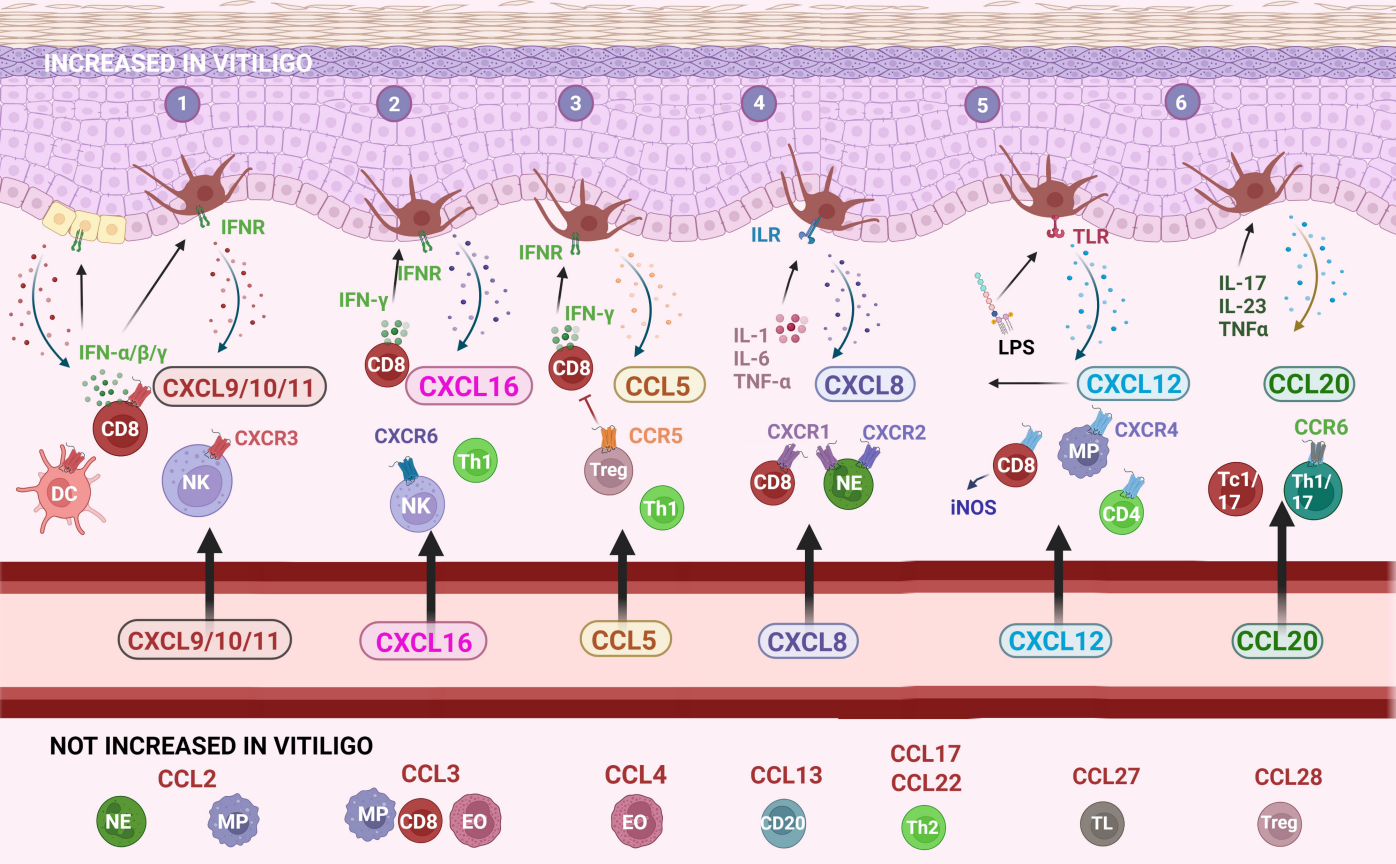
The main chemokines and receptors and their recruitment of immune cells into vitiligo skin. (1) CXCL9/10/11 chemokines bind to the CXCR3 receptor that plays a key role in the development of a cytotoxic CD8 response *via* IFN-γ; (2) CXCL16-CXCR6 signaling recruits NK cells, CD8 and CD4 lymphocytes; (3) the CCL5-CCR5 pathway attracts a broader range of immune cells into the skin; (4) CXCL8 is mostly recognized for its effect on neutrophils although a subset of CD8 lymphocytes also responds to this chemokine; (5) CXCL12 recruits several immune cells including macrophages and NK cells and also stimulates melanocyte migration; (6) CCL20 possibly involved in the recruitment of Tc1/17 and Th1/17 towards the skin. CCL: chemokine (C-C motif) ligand; CCR: chemokine (C-C motif) receptor; CD: cluster of differentiation; CXCL: chemokine (C-X-C motif) ligand; CXCR: chemokine (C-X-C motif) receptor; DC: dendritic cell; EO: eosinophil; IFN: interferon; IFNR: interferon receptor; IL: interleukin; ILR: interleukin receptor; iNOS: inducible nitric oxide synthase; LPS: lipopolysaccharides; MP: macrophage/monocyte; NE: neutrophil; NK: natural killer cell; Tc: cytotoxic T lymphocyte; Th: T helper lymphocyte; TL: skin-homing T lymphocyte; TLR: Toll-like receptor; TNF: tumor-necrosis factor; Treg: regulatory T cells. Created with Biorender.com.

CCL5 is a strong recruiting cytokine of NK cells, eosinophils, monocytes, effector memory T cells, B cells, and immature dendritic cells (**Figure 2**). CCL5 is induced by IFN-γ. 3/5 studies (266 patients versus 111 controls) reported significantly increased CCL5 concentrations in the circulation of vitiligo patients compared to healthy controls. The overall value of the meta-analysis was significant (P = 0.0008), although this result has to be interpreted with caution given the high variability in results ranging from no significant difference to an 11-fold difference in CCL5 levels (13, 20, 22, 33, 40). 3 studies investigated whether CCL5 was linked to disease activity with 2 studies documenting no significant difference and 1 report pointing to a small but significantly higher CCL5 concentration in active vitiligo (1.23-fold higher).

Yang et al., 2018 found increased CCL5 levels in the blister fluid of vitiligo patients compared to controls and higher values in active vitiligo versus stable vitiligo (13). Rashighi et al., 2014 detected increased CCL5 expression in lesional vitiligo skin compared to healthy controls (23). RNA analysis showed that *in vitro* cultured vitiligo melanocytes display a 23.5-fold upregulation of CCL5 (36). CCL5 can bind to CCR1, CCR3, and CCR4, although its highest affinity is with CCR5 (41). Gellatly et al., 2021 found that CCL3/4/5-CCR5 interaction is one the most affected pathways in vitiligo skin. CCR5 is upregulated on regulatory T cells (Tregs) in vitiligo promoting its function to suppress CD8+T cells (42).

### CXCR1 and CXCR2 pathway: CXCL8

CXCL8 (= IL-8) is a proinflammatory cytokine expressed by a variety of cell types such as monocytes, macrophages, fibroblasts, endothelial, and epithelial cells including melanocytes (35). 3 studies (264 patients and 88 healthy controls) reported higher circulating CXCL8 values, although only one was statistically significant (13, 15, 22). A meta-analysis demonstrated also a significant overall effect (P = 0.03). CXCL8 levels were higher in active compared to stable vitiligo in 2 studies (P = 0.002). In one study, higher concentrations of CXCL8 were present in the blister fluid of vitiligo patients compared to healthy controls with increasing values in patients with progressive disease (13).

Vitiligo melanocytes seem to produce more CXCL8 compared to normal melanocytes (36). CXCL8 is produced upon stimulation of cells by different cytokines (e.g., IL-1, IL-6, and TNF-α) but also by other chemokines (e.g., CXCL12), reactive oxygen species, environmental stress, and bacteria. CXCL8 binds to CXCR1 and CXCR2, resulting in the chemotaxis and activation of neutrophils (43). CXCR1+ CD8 cells responding to CXCL8 have been linked with immediate cytotoxicity and early migration after innate immune activation (44). CXCL8 induces an oxidative burst, enhancing further inflammatory responses. It also induces endothelial cell permeability and angiogenesis (45). Unsurprisingly, this chemokine has been implicated in a variety of inflammatory disorders including psoriasis, asthma, inflammatory bowel syndrome, cystic fibrosis, and chronic obstructive pulmonary disorder (43).

## Chemokine at the crossroads of Th17 and Th1 responses

### CCL20/CCR6 receptor

Zhang et al., 2019 documented increased serum CCL20 values in vitiligo vs healthy controls and found slightly higher values depending on disease activity (46). Additionally, CCL20 concentrations were increased in the blister fluid of vitiligo patients compared to controls. CCL20 is induced by IL-17A, IL-23, and TNF-α and secreted by keratinocytes (46). The CCL20-CCR6 axis attracts IL-17A-producing cells to the skin and is elevated in chronic inflammatory diseases including psoriasis, inflammatory bowel disease and arthritis (46). However, the authors hypothesized that CCL20 may be involved in the recruitment of dual producing IL-17 and IFN-γ cells (CCR6+ Th1/17 cells and Tc1/17 cells) to vitiligo skin (46). Another study confirmed higher serum CCL20 concentrations in patients with active disease (47). In contrast, Rashighi et al., 2014 did not found increased CCL20 expression in lesional vitiligo skin versus controls (n=5 per group) (23). CCL20/CCR6 signaling recruits also Tregs to the skin and Tregs lacking CCR6 have a decreased capacity to migrate to vitiligo skin and suppress depigmentation (48).

## Chemokines attracting neutrophils

### CCL2/CCR2 receptor signaling

CCL2 is involved in the Th2 response and regulated by IL-4. CCL2 mainly attracts neutrophils and monocytes (49). CCL2 and IL-6 promote the survival of myeloid monocytes and trigger the differentiation towards M2-type macrophages (50). Additionally LPS-activated melanocytes have a high expression of CCL2 (35). No difference in serum CCL2 concentrations were found in vitiligo (170 vitiligo vs 90 control) (22, 28). Similarly, no higher values were found in progressive vitiligo (22).

## Other chemokines

Several chemokines have to date only been investigated in one study and lack confirmation data.

### Elevated chemokines without confirmatory data

Serum CCL3 levels were also higher in vitiligo patients although no link with disease activity was found (15). Nonetheless, a decreased production of CCL3 by vitiligo melanocytes has been reported (36). CCL3 is produced by a variety of hematopoietic and non-hematopoietic cells and recruits macrophages, eosinophils, and lymphocytes *via* the CCR1 or CCR5 receptor with preferential activity on activated CD8+ T cells (51). CCL3 was upregulated in CD8+ T cells of lesional and non-lesional vitiligo skin (42). Proteomic profiling of plasma (28 vitiligo patients versus 28 controls) showed elevated CXCL4 and CXCL7 levels (52). Higher CXCL4 levels would be in line with the increased CXCR3 signaling found in vitiligo.

### Non-elevated chemokines without confirmatory data

No significantly elevated values were detected for CCL1, CXCL1, CCL4, CXCL13, CCL17, CCL22, CCL27, and CCL28 in the circulation of vitiligo patients. Most of these chemokines act on innate immune cells (e.g. neutrophils or eosinophils: CXCL1, CCL28), Th2 lymphocytes (CCL4, CCL17, CCL22), B lymphocytes (CXCL13), or Tregs (CCL28) (**Figure 2**). These cell types are therefore less likely to play a crucial role in the pathogenesis of vitiligo.

## Conclusion

A substantial number of studies have confirmed the specific chemokine profile associated with vitiligo. Prominent CXCR3 signaling has been uncovered by the increased expression of CXCL9, CXCL10, CXCL11, and CXCL4. Of these chemokines, CXCL10 seems to display the most notable change in the blood circulation of vitiligo patients and is elevated in patients with active disease. In contrast, the levels of CXCL9 and CXCL11 tend to be more variable. The relevance of the CXCL12-CXCR4 pathway in vitiligo has also been confirmed although its differential expression in the skin has only been investigated to a limited extent. CXCL12 induces CXCL8 production which is also more pronounced in vitiligo patients. CCL5-CCR5 expression is elevated in vitiligo patients compared to healthy controls although a link with disease activity could not be confirmed. The CXCL16-CXCR6 pathway is another important pathogenic mechanism driving the progression of vitiligo by the recruitment of CD8+ lymphocytes (20). Most vitiligo studies have concentrated on a small number of chemokines based on previous findings, as evidenced by the large number of studies on CXCL9 and CXCL10. This method runs the risk of leaving key chemokines undiscovered for the time being. A possible role for CXCL4, CXCL7, and CCL18 has been suggested (42, 52). Broad chemokine profile studies are advisable to offer a non-biased approach.

## Author contributions

RS performed the literature search, meta-analysis and wrote the draft of the article. AB created **Figure 2**. AB, MS, and NvG did a critical review of the manuscript. All authors contributed to the article and approved the submitted version.

## Supplementary material

Supplementary Figure 1 PRISMA flow diagram.

Supplementary Figure 2 Meta-analysis of chemokines at the protein level in skin blister fluid of vitiligo skin.
