# Supplementary figures and images for: A meta-analysis of chemokines in vitiligo: Recruiting immune cells towards melanocytes
Source: Front Immunol. 2023 Feb 24;14:1112811. doi: 10.3389/fimmu.2023.1112811 (PMC9999440; doi:10.3389/fimmu.2023.1112811)

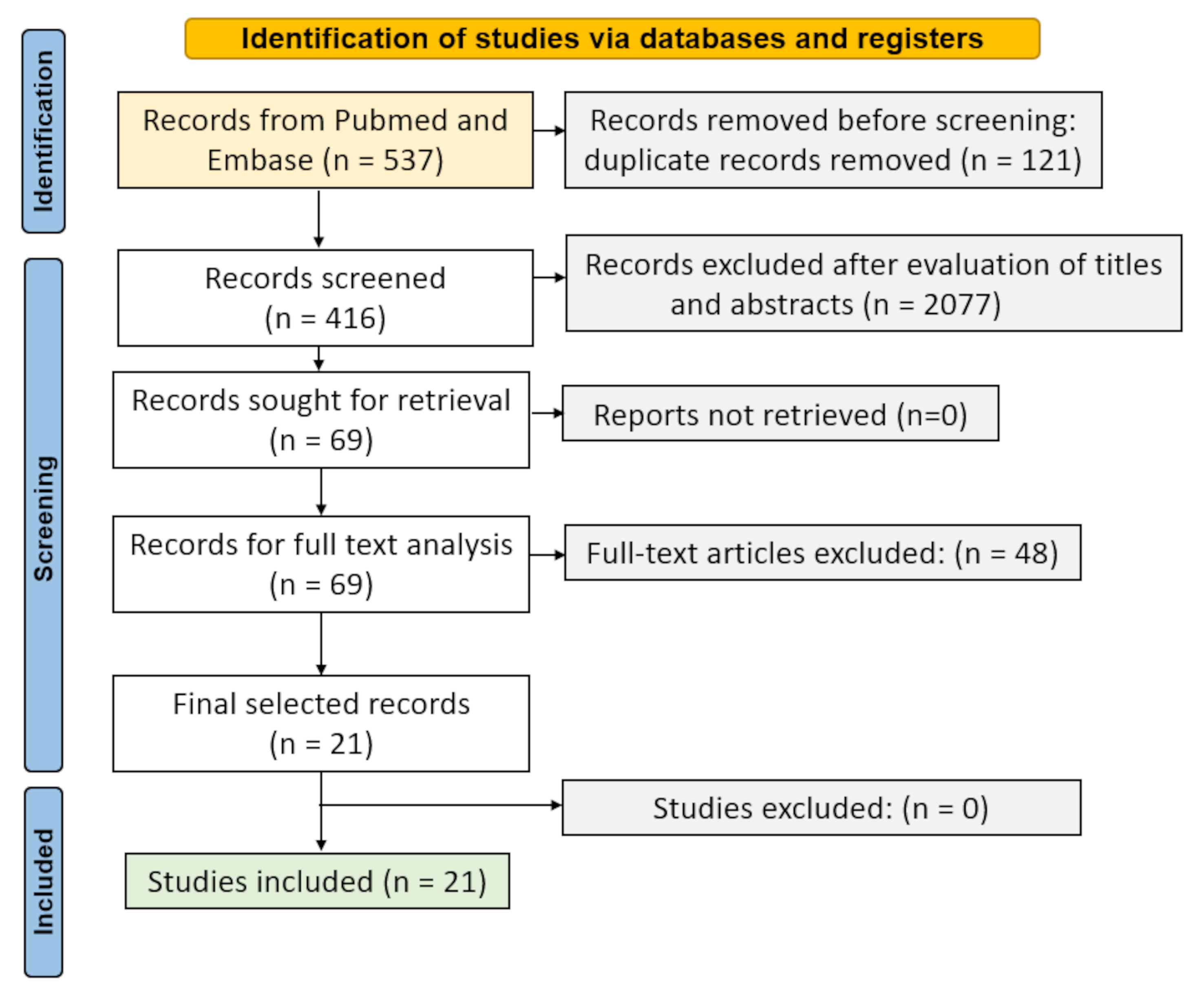

Supplement: Supplementary Figure 1 — PRISMA flow diagram. [file Image_1.tiff]

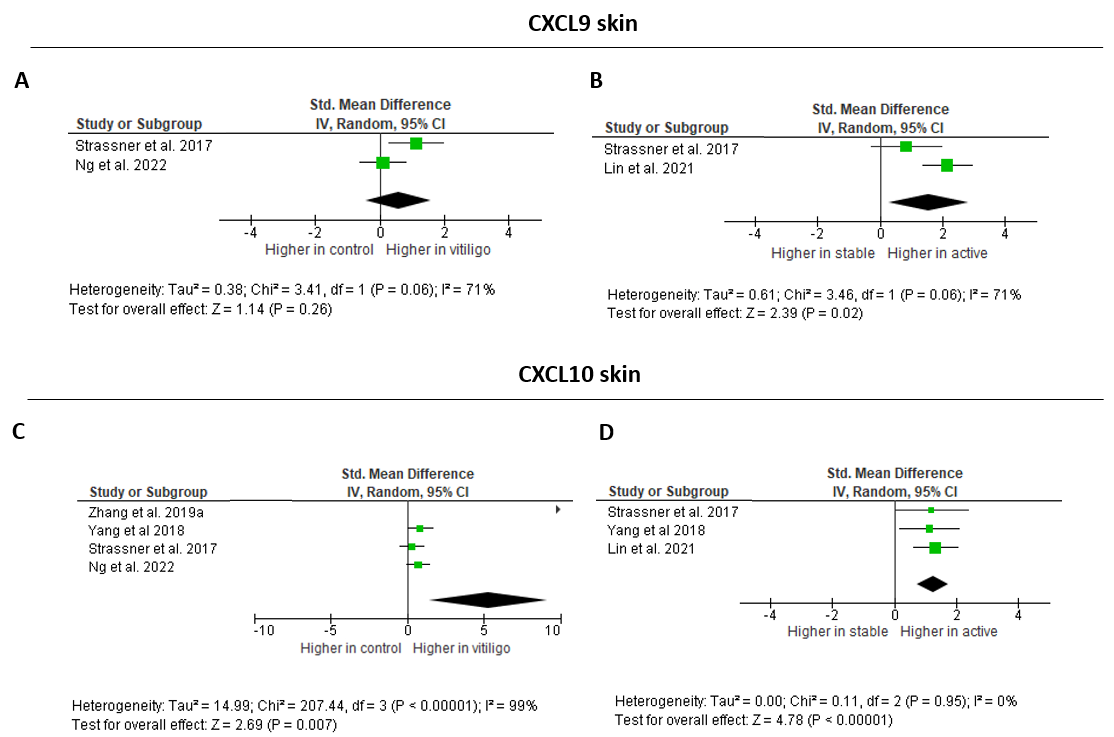

Supplement: Supplementary Figure 2 — Meta-analysis of chemokines at the protein level in skin blister fluid of vitiligo skin. [file Image_2.tiff]
